# Adjuvant Sorafenib Following Radiofrequency Ablation for Early-Stage Recurrent Hepatocellular Carcinoma With Microvascular Invasion at the Initial Hepatectomy

**DOI:** 10.3389/fonc.2022.868429

**Published:** 2022-06-23

**Authors:** Meng-Chao Wei, Yao-Jun Zhang, Min-Shan Chen, Yong Chen, Wan-Yee Lau, Zhen-Wei Peng

**Affiliations:** ^1^ Department of Liver Surgery, Cancer Center, Sun Yat-sen University, Guangzhou, China; ^2^ Department of Surgery, Peking Union Medical College Hospital, Chinese Academy of Medical Science and Peking Union Medical College, Beijing, China; ^3^ State Key Laboratory of Oncology in South China, Guangzhou, China; ^4^ Department of Radiation Oncology, The First Affiliated Hospital, Sun Yat-sen University, Guangzhou, China; ^5^ Faculty of Medicine, The Chinese University of Hong Kong, Prince of Wales Hospital, Shatin, Hong Kong, Hong Kong SAR, China; ^6^ The Institute of Precision Medicine, The First Affiliated Hospital, Sun Yat-sen University, Guangzhou, China

**Keywords:** recurrent hepatocellular carcinoma, microvascular invasion, sorafenib, radiofrequency ablation, adjuvant therapy

## Abstract

**Background:**

The efficacy of radiofrequency ablation (RFA) for patients with early-stage recurrent hepatocellular carcinoma (HCC) with microvascular invasion (MVI) at the initial hepatectomy is limited. Our study aimed to explore whether adjuvant sorafenib following RFA could improve the situation.

**Methods:**

We retrospectively included 211 patients with early-stage (tumor number of ≤3 and tumor size of 2–5 cm) recurrent HCC with MVI at the initial hepatectomy who underwent adjuvant sorafenib following RFA or RFA alone in 13 centers from June 2013 to June 2020. In the combination group, sorafenib of 400 mg twice daily was administered within 7 days after RFA. Overall survival (OS) and recurrence-free survival (RFS) were compared. Subgroup analysis based on MVI grade was performed. MVI grade was based on the practice guidelines for the pathological diagnosis of HCC and included M1 (≤5 MVI sites, all located within adjacent peritumoral liver tissues 0–1 cm away from the tumor margin) and M2 (>5 MVI sites, or any MVI site located within adjacent peritumoral liver tissues > 1 cm away from the tumor margin).

**Results:**

A total of 103 patients received the combination therapy and 108 patients received RFA alone. The combination therapy provided better survival than RFA alone (median RFS: 17.7 vs. 13.1 months, *P* < 0.001; median OS: 32.0 vs. 25.0 months, *P* = 0.002). Multivariable analysis revealed that treatment allocation was an independent prognostic factor. On subgroup analysis, the combination therapy provided better survival than RFA alone in patients with M1 along with either a tumor size of 3–5 cm, tumor number of two to three, or alpha-fetoprotein (AFP) > 400 μg/L, and in those with M2 along with either a tumor size of 2–3 cm, one recurrent tumor, or AFP ≤ 400 μg/L.

**Conclusions:**

Adjuvant sorafenib following RFA was associated with better survival than RFA alone in patients with early-stage recurrent HCC with MVI at the initial hepatectomy. Moreover, MVI grade could guide the application of adjuvant sorafenib.

## Introduction

Nearly 70% of patients with early-stage hepatocellular carcinoma (HCC) develop recurrence within 5 years following hepatectomy (1). Repeated hepatectomy and salvage liver transplantation are effective treatments for HCC recurrence (2). However, the wide application of these two strategies is limited due to poor liver functional reserve following initial hepatectomy and liver donor shortage for transplantation.

Radiofrequency ablation (RFA) has shown similar survival outcomes to repeated hepatectomy in treating early-stage recurrent HCC following hepatectomy (3). However, RFA presented worse survival than repeated hepatectomy in patients with aggressive recurrent HCC, including those with a tumor size greater than 3 cm (3–5), an alpha-fetoprotein (AFP) level greater than 200 μg/L (3), and who relapsed within 2 years following initial resection (5). Therefore, it is significant to enhance the efficacy of RFA in patients with aggressive early-stage recurrent HCC.

Microvascular invasion (MVI) is associated with poor tumor differentiation, aggressive behavior, and worse survival outcomes in recurrent HCC (6). Previous studies have investigated RFA for patients with early-stage recurrent HCC with MVI at the initial hepatectomy (6–8). These studies integrated repeated hepatectomy and RFA as one curative group. The survival outcomes of the curative treatments were limited, even inferior to transarterial chemoembolization (TACE) (8). Therefore, more effort should be made to enhance the efficacy of RFA in patients with early-stage recurrent HCC with MVI at the initial hepatectomy.

Sorafenib was once the first-line systemic therapy for advanced HCC (9, 10). Several studies have shown the combination of sorafenib, and RFA is associated with a lower incidence of post-RFA recurrence and better survival than RFA alone in treating primary or recurrent HCC (11–13), indicating the important role of sorafenib in enhancing the efficacy of RFA. For instance, Feng et al. evaluated the efficacy of combined sorafenib and RFA in 64 patients with HCC at Barcelona Clinic Liver Cancer group (BCLC) stage 0–B1, of which 48 were recurrent, and sorafenib was administered after RFA in 54 patients. The combination therapy exhibited a 4-year overall survival (OS) rate of 50.3%, significantly better than 30.9% in the RFA-alone group (11). Moreover, as an angiogenesis inhibitor, sorafenib has exhibited significant survival benefit as an adjuvant therapy following curative hepatectomy in patients with MVI-positive HCC (14, 15). Nevertheless, there has been no published evidence on applying sorafenib following RFA in patients with early-stage recurrent HCC with MVI at the initial hepatectomy.

Therefore, our study aimed to determine the role of adjuvant sorafenib following RFA in patients with early-stage recurrent HCC with MVI at the initial hepatectomy, with an attempt to improve the present situation of applying RFA in patients with high-risk early-stage recurrent HCC.

## Materials and Methods

### Study Design and Patients

This is a retrospective multicentric study conducted in 13 medical centers in China, namely Anhui Provincial Hospital, Beijing Cancer Hospital, the First and the Third Department of Shanghai Eastern Hepatobiliary Surgery Hospital, Fudan Zhongshan Hospital, Cancer Center of Sun Yat-sen University, Bethune First Hospital of Jilin University, Tianjin Medical University Cancer Hospital, Xijing Hospital, Cancer Hospital Chinese Academy of Medical Sciences, The First Affiliated Hospital of Zhejiang University, The First Affiliated Hospital of Zhengzhou University, and the Southwest Hospital of AMU. The study was approved by all the Ethics Committees of the individual centers, and it conformed to the standards of the Declaration of Helsinki. Informed consent was waived because of the retrospective design of the study.

From June 2013 to June 2020, 21,912 consecutive patients were diagnosed with intrahepatic recurrences after R0 liver resection for HCC according to the non-invasive criteria of the American Association for the Study of Liver Diseases (16). HCC with MVI positivity was diagnosed in the resected liver specimens in 1,312 patients. In each institution, MVI at the first resection was confirmed by two experienced pathologists in hepatology over 5 years. The inclusion criteria were as follows: (1) age between 18 and 75 years; (2) first intrahepatic recurrence after R0 hepatectomy; (3) early-stage recurrent HCC with tumor number of ≤3 and tumor size of 2–5 cm; (4) absence of macrovascular invasion or extrahepatic metastasis; (5) Child-Pugh Class A-B; (6); adequate hematologic and renal function as previously described (17); (7); an Eastern Cooperative Oncology Group (ECOG) performance score of 0; and (8) the duration of sorafenib treatment was at least 3 months in the combination group. Patients with a history of another malignancy, associated severe organic dysfunction, or previous or concomitant systemic anti-cancer treatments were excluded. The therapeutic selection between the combination therapy and RFA alone was made by a multidisciplinary team consisting of specialists from hepatic surgery, interventional radiology, and oncology, based on tumor characteristics and liver function, as well as patients’ willingness. For example, patients with high-risk factors for recurrence including larger tumor size or more tumor lesions may be recommended to receive the combined therapy, whereas patients with earlier tumor stage of primary HCC or worse liver function may be recommended to receive RFA alone.

### RFA Procedure and Sorafenib Administration

In each institution, percutaneous RFA was performed by two interventional clinicians with over 10 years of RFA experience under real-time ultrasound guidance as previously reported (18). Treatment was performed under moderate sedation and local anesthesia. A commercially available Cool-tipTM RFA system (Valleylab, Boulder, CO, USA) with a needle of 3-cm active tip length was used. The needle was inserted into the tumor under ultrasound guidance, aiming to generate an ablative zone covering an area larger than 1 cm around the tumor. The number of needle punctures and ablation points was determined by tumor size. The multiple-overlapping technique was applied for each tumor. The needle tract was ablated at the end of the procedure to prevent bleeding and tumor seeding. Technical success of ablation was evaluated by contrast-enhanced ultrasound (CEUS) 1 month after RFA. If residual unablated tumor was detected, then additional RFA was performed.

For patients who received the combination of sorafenib and RFA, sorafenib was administered orally at a dosage of 400 mg twice daily. The drug was administered within 7 days following RFA based on the liver function status. For limited toxicity, the administration regimen was modified to 200 mg twice daily or 400 mg on alternate days, but the drug was discontinued if severe toxicity occurred.

### Follow-Up

Routine contrast-enhanced computed tomography (CECT) and CEUS were performed 4 weeks after RFA to assess treatment effectiveness. The patients were then followed-up once every 3 months for the first 2 years and once every 6 months thereafter. At each follow-up visit, clinical evaluation, CEUS, liver function tests, and AFP were performed. CECT or magnetic resonance imaging was performed once every 6 months. Chest CT and bone scintigraphy were performed when extrahepatic metastasis was clinically suspected. When local tumor progression and intrahepatic or extrahepatic recurrence were diagnosed, patients were offered treatments, which included repeated hepatectomy, RFA, TACE, sorafenib (only in the RFA-alone group), levatinib, apatinib, immunotherapy, or the best supportive care according to the number and size of recurrent tumors and liver function.

### Outcomes

Adverse events were evaluated by the National Cancer Institute Common Toxicity Criteria Grading version 4.0. Severe adverse events (grade ≥3) were defined as clinical events requiring additional therapeutic interventions or prolonged hospitalization (19). OS was defined as the time interval between the initial diagnosis of recurrent HCC and the date of death or the last follow-up. Recurrence-free survival (RFS) was defined as the time interval between the initial diagnosis of recurrent HCC and the date of HCC re-recurrence or the last follow-up. The study was censored on December 31, 2020.

### Statistical Analysis

Continuous variables were presented as mean ± SD. Categorical variables were presented as numbers and percentages. Difference test was conducted using t-test for continuous variables and χ^2^ test or Fisher’s exact test for categorical variables. Patients’ characteristics, including age, sex, hepatitis B surface antigen (HBsAg), tumor size, tumor number, platelet (PLT), albumin (ALB), alanine aminotransferase (ALT), total bilirubin (TBIL), prothrombin activity, AFP, BCLC stage of primary HCC, interval of recurrence from initial treatment, initial hepatic resection type, antiviral treatment for hepatitis B, and MVI grade of primary HCC were analyzed by univariable and multivariable Cox proportional hazard regression models to identify potential survival predictors. Of note, MVI grade was based on the practice guidelines for the pathological diagnosis of primary liver cancer (20). M1 represents low-risk with MVI of ≤5 sites and all located within adjacent peritumoral liver tissues 0–1 cm away from the tumor margin, and M2 stands for high-risk with MVI of >5 sites, or any MVI site located within adjacent peritumoral liver tissues > 1 cm away from the tumor margin. Survival curves were generated by the Kaplan–Meier method and compared by the log-rank test. To elaborate the role of MVI grade in the treatment of recurrent HCC, subgroup analysis based on significant survival predictors was performed in patients with different MVI grades. Statistical analysis was conducted using SPSS software (version 20.0, SPSS Inc., Chicago, IL). All tests were two-sided, and *P* < 0.05 indicated statistical significance.

## Results

### Patient Characteristics

**Figure 1:**
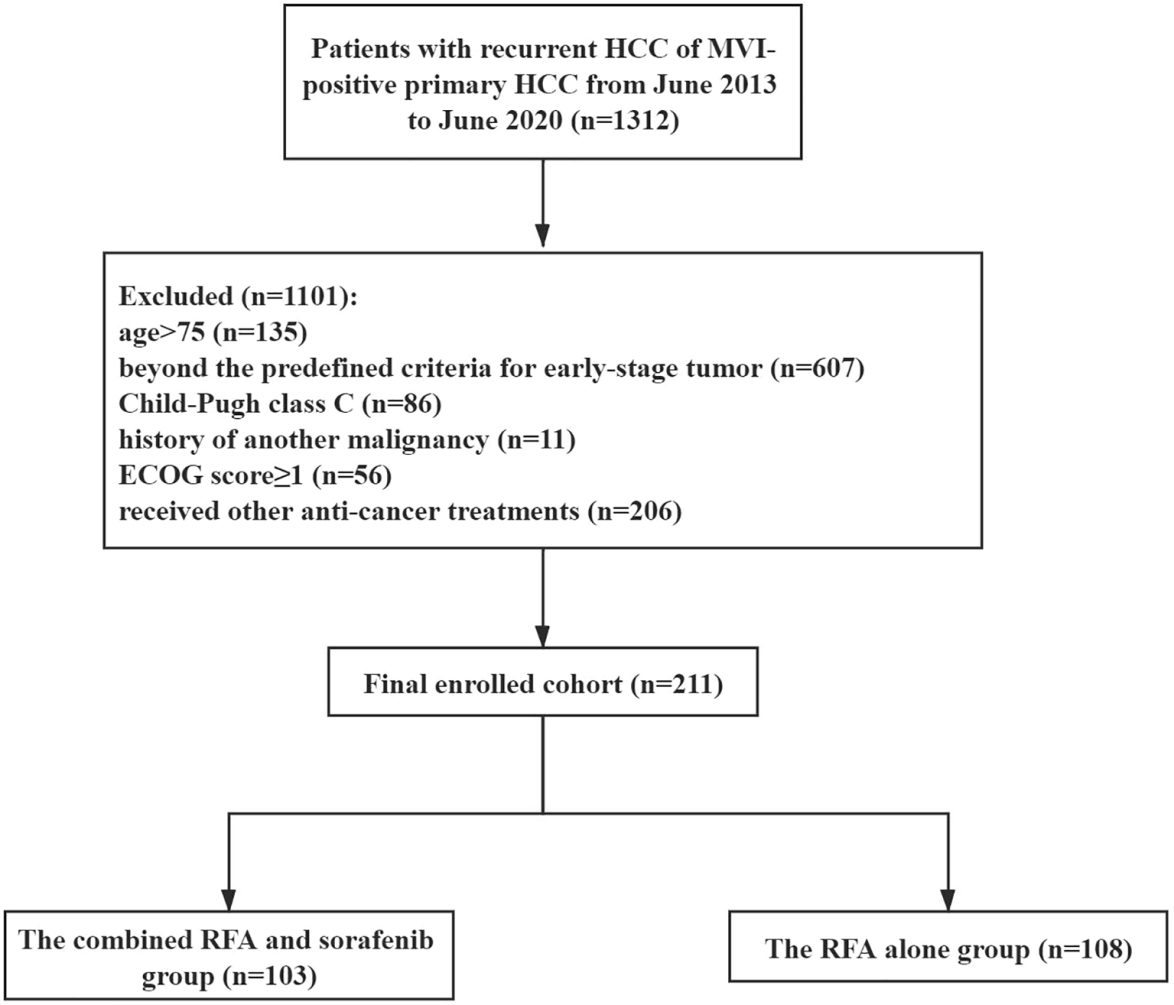
The flow chart of patient enrollment. HCC, hepatocellular carcinoma; MVI, microvascular invasion; ECOG, Eastern Cooperative Oncology Group; RFA, radiofrequency ablation.

The flow chart of patient enrollment was shown in **Figure 1**. We finally enrolled 103 patients in the combined RFA and sorafenib group (mean age, 54 ± 6 years; 86 men) and 108 patients in RFA-alone group (mean age, 53 ± 9 years; 94 men). The baseline characteristics were summarized in **Table 1**. All the listed variables were comparable between the two groups (all *P* > 0.05).

**Table 1:** Baseline characteristics of the two treatment groups.

Variable	RFA-Sorafenib (n = 103)	RFA (n = 108)	*P*-value
Age (year) (range)	54 ± 6	53 ± 9	0.139
Sex (man/woman)	86 (83.5%) /17 (16.5%)	94 (87.0%) /14 (13.0%)	0.468
HBsAg (+/−)	95 (92.2%) /8 (7.8%)	102 (94.4%) /6 (5.6%)	0.519
Tumor size (cm) (2–3/3–5)	50 (48.5%) /53 (51.5%)	51 (47.2%) /57 (52.8%)	0.848
Tumor number (1/2–3)	63 (61.2%) /40 (38.8%)	57 (52.8%) /51 (47.2%)	0.219
PLT (×10^9^/L)	102.7 ± 35.6	112.0 ± 25.7	0.095
ALB (g/L)	35.5 ± 2.1	35.3 ± 3.2	0.875
ALT (U/L)	31.2 ± 6.8	29.3 ± 14.6	0.101
TBIL (μmol/L)	9.8 ± 4.7	8.9 ± 6.5	0.561
Prothrombin activity (%)	89.6 ± 15.6	91.6 ± 13.2	0.382
AFP (μg/L) (≤ 400/>400)	62 (60.2%) /41 (39.8%)	65 (60.2%) /43 (39.8%)	0.999
Tumor stage of primary HCC (BCLC A/B)	84 (81.6%) /19 (18.4%)	96 (88.9%) /12 (11.1%)	0.132
Interval of recurrence from initial treatment (year)			0.649
≤1	54 (52.4%)	60 (55.6%)	
>1	49 (47.6%)	48 (44.4%)	
Initial hepatic resection type			0.403
One segment	64 (62.1%)	61 (56.5%)	
More than one segments	39 (37.9%)	47 (43.5%)	
Antiviral treatment for hepatitis B (yes/no)	72 (69.9%) /31 (30.1%)	80 (74.1%) /28 (25.9%)	0.500
MVI grade (M1/M2)	59 (57.3%) /44 (42.7%)	59 (54.6%) /49 (45.4%)	0.698

RFA, radiofrequency ablation; HBsAg, hepatitis B surface antigen; PLT, platelet; ALB, albumin; ALT, alanine aminotransferase; TBIL, total bilirubin; AFP, alpha-fetoprotein; HCC, hepatocellular carcinoma; BCLC, Barcelona Clinic Liver Cancer group; MVI, microvascular invasion.

### Efficacy

**Figure 2:**
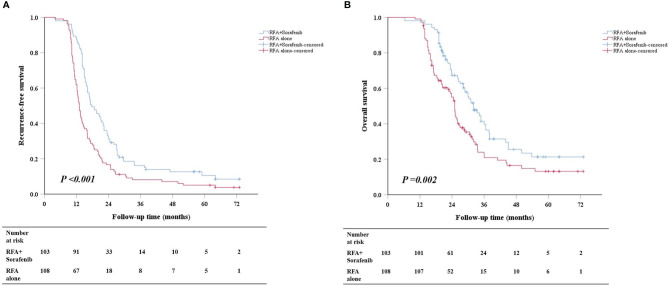
Cumulative survival curves of RFS **(A)** and OS **(B)** between the combination group and RFA-alone group in the whole cohort. RFS, recurrence-free survival; OS, overall survival; RFA, radiofrequency ablation.

The mean ± SD follow-up time was 39.3 ± 12.1 months for the combination group and 38.4 ± 12.6 months for RFA-alone group. The numbers of patients who received modification, discontinuation, and withdrawal of sorafenib in the combination group were 72, 7, and 3, respectively. Survival analysis revealed that the combination therapy provided better survival than RFA alone (median RFS: 17.7 vs. 13.1 months, *P* < 0.001; median OS: 32.0 vs. 25.0 months, *P* = 0.002) (**Figures 2A, B**). In patients with M1, the RFS of the combination group was longer than that of RFA-alone group (median RFS: 18.7 vs. 14.0 months, *P* = 0.013) (**Supplemental Figure 1A**); however, the OS was similar between the two groups (median OS: 33.4 vs. 25.5 months, *P* = 0.102) (**Supplemental Figure 1B**). Meanwhile, in patients with M2, both RFS and OS of the combination group were superior to those of RFA-alone group (median RFS: 17.2 vs. 12.5 months, *P* < 0.001; median OS: 28.8 vs. 22.5 months, *P* = 0.004) (**Supplemental Figures 2A, B**).

### Univariable and Multivariable Analysis

Univariable and multivariable analysis showed that tumor size [3–5 cm vs. 2–3 cm, hazard ratio (HR) =1.526, 95% confidence interval (CI): 1.140–2.044, *P* = 0.005], tumor number (2–3 vs. 1, HR =1.485, 95% CI: 1.092–2.011, *P* = 0.015], PLT (>100 × 10^9^/L vs. ≤100 × 10^9^/L, HR = 2.296, 95% CI: 1.151–4.582, *P* = 0.018], AFP (>400 μg/L vs. ≤400 μg/L, HR = 2.150, 95% CI: 1.587–2.911, *P* < 0.001), interval of recurrence from initial treatment (>1 years vs. ≤1 year, HR = 0.641, 95% CI: 0.465–0.883, *P* = 0.006), MVI grade (M2 vs. M1, HR = 1.695, 95% CI: 1.251–2.295, *P* = 0.001), and treatment allocation (RFA vs. combination therapy, HR =1.956, 95% CI: 1.439-2.658, *P* < 0.001) were independent prognostic factors of RFS, whereas tumor size (3–5 cm vs. 2–3 cm, HR = 1.715, 95% CI: 1.217–2.416, *P* = 0.002), tumor number (2–3 vs. 1, HR = 1.744, 95% CI: 1.181–2.590, *P* = 0.004), PLT (>100 × 10^9^/L vs. ≤100 × 10^9^/L, HR = 3.563, 95% CI: 1.665–7.625, *P* = 0.001), AFP (>400 μg/L vs. ≤400 μg/L, HR = 2.287, 95% CI: 1.615–3.238, *P* < 0.001), MVI grade (M2 vs. M1, HR = 1.623, 95% CI: 1.111–2.139, *P* = 0.007), and treatment allocation (RFA vs. combination therapy, HR = 1.636, 95% CI: 1.129–2.370, *P* = 0.009) were independent prognostic factors of OS (**Table 2**).

**Table 2:** Univariable and multivariable analysis of prognostic factors.

Variables^†^	Recurrence-Free Survival						Overall Survival					
	Univariable Analysis			Multivariable Analysis			Univariable Analysis			Multivariable Analysis		
	HR	95% CI	*P*-value	HR	95% CI	*P-*value	HR	95% CI	*P*-value	HR	95% CI	*P-*value
Age [ ≤ 60 years]	0.842	0.630–1.125	0.245				0.803	0.573–1.126	0.204			
Sex [man]	0.989	0.666–1.468	0.956				0.884	0.539–1.450	0.626			
HBsAg [−]	0.570	0.318–1.025	0.060				0.351	0.143–0.857	0.022^*^	0.432	0.173–1.081	0.079
Tumor size [2–3 cm]	1.461	1.099–1.942	0.009^*^	1.526	1.140–2.044	0.005^*^	1.544	1.108–2.150	0.010^*^	1.715	1.217–2.416	0.002^*^
Tumor number [1]	1.655	1.239–2.211	0.001^*^	1.485	1.092–2.011	0.015^*^	1.864	1.337–2.599	< 0.001^*^	1.744	1.181–2.590	0.004^*^
PLT [ ≤ 100 × 10^9^/L]	2.245	1.139–4.423	0.019^*^	2.296	1.151–4.582	0.018^*^	3.223	1.562–6.652	0.002^*^	3.563	1.665–7.625	0.001^*^
ALB [ ≤ 35 g/L]	1.302	0.980–1.731	0.069				1.578	1.136–2.192	0.007^*^	0.863	0.529–1.410	0.557
ALT [ ≤ 40 U/L]	1.016	0.756–1.367	0.914				1.124	0.800–1.579	0.499			
TBIL [ ≤ 20.5 μmol/L]	1.422	1.041–1.941	0.027^*^	1.183	0.847–1.653	0.324	1.130	0.795–1.608	0.495			
Prothrombin activity [ ≤ 70%]	1.277	0.957–1.702	0.097				1.592	1.146–2.212	0.006^*^	1.579	0.966–2.580	0.068
AFP [≤ 400 μg/L]	1.886	1.413–2.516	< 0.001^*^	2.150	1.587–2.911	< 0.001^*^	2.172	1.559–3.027	< 0.001^*^	2.287	1.615–3.238	< 0.001^*^
Tumor stage of primary HCC [BCLC A]	1.105	0.748–1.633	0.615				1.150	0.736–1.798	0.539			
Interval of recurrence from initial treatment [ ≤ 1 year]	0.658	0.493–0.879	0.005^*^	0.641	0.465–0.883	0.006^*^	0.650	0.466–0.909	0.012^*^	0.728	0.497–1.068	0.104
Initial hepatic resection type [one segment]	1.076	0.807–1.434	0.618				1.347	0.970–1.871	0.076			
Antiviral treatment for hepatitis B [yes]	0.886	0.644–1.218	0.455				0.862	0.592–1.253	0.436			
MVI grade [M1]	1.512	1.136–2.012	0.005^*^	1.695	1.251–2.295	0.001^*^	1.528	1.099–2.126	0.012^*^	1.623	1.111–2.139	0.007^*^
Treatment allocation [combination therapy]	1.749	1.315–2.325	< 0.001^*^	1.956	1.439–2.658	< 0.001^*^	1.670	1.199–2.327	0.002^*^	1.636	1.129–2.370	0.009^*^

*Statistically significant at alpha = 0.05. ^†^Data in square brackets is the reference.

HR, hazard ratio; CI, confidence interval; HBsAg, hepatitis B surface antigen; PLT, platelet; ALB, albumin; ALT, alanine aminotransferase; TBIL, total bilirubin; AFP, alpha-fetoprotein; HCC, hepatocellular carcinoma; BCLC, Barcelona Clinic Liver Cancer group; MVI, microvascular invasion.

### Subgroup Analysis

On the basis of significant survival predictors including tumor size, tumor number, and AFP, we performed subgroup analysis in patients with different MVI grades. The median survival of the combination group and RFA-alone group along with the HRs of the combination therapy in different subgroups is summarized in **Figures 3A, B**. The detailed survival curves were shown in **Supplemental Figures 3–14**.

**Figure 3:**
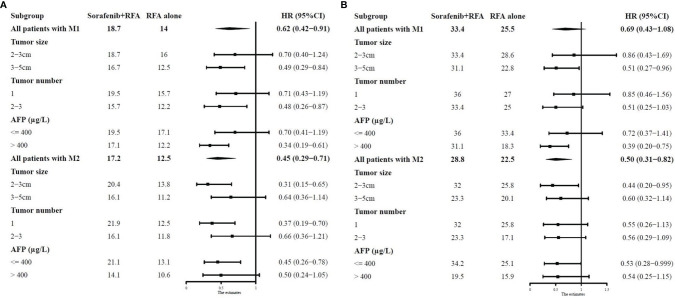
Forest plots showing the median RFS **(A)** and OS **(B)** of the combination group and RFA-alone group along with the HRs of the combination therapy in different subgroups. RFS, recurrence-free survival; OS, overall survival; RFA, radiofrequency ablation; HR, hazard ratio; CI, confidence interval; AFP, alpha-fetoprotein.

In patients with M1, the survival rates were similar between the two treatment groups in the 2- to 3-cm subgroup (RFS, *P* = 0.215; OS, *P* = 0.650). In contrast, the combination therapy exhibited superior survival rates than RFA alone in the 3- to 5-cm subgroup (RFS, *P* = 0.007; OS, *P* = 0.031). For patients with one recurrent tumor, the survival rates were similar between the two treatment groups (RFS, *P* = 0.185; OS, *P* = 0.596). Meanwhile, for patients with two to three recurrent tumors, the combination group had better RFS and similar OS than RFA-alone group (RFS, *P* = 0.013; OS, *P* = 0.052). In the subgroup of AFP ≤400 μg/L, the survival rates were similar between the two treatment groups (RFS, *P* = 0.180; OS, *P* = 0.335). However, in the subgroup of AFP >400 μg/L, the combination group was superior to RFA-alone group in terms of both RFA and OS (RFS, *P* < 0.001; OS, *P* = 0.003).

In patients with M2, the combination therapy exhibited superior survival rates than RFA alone in the 2- to 3-cm subgroup (RFS, *P* = 0.001; OS, *P* = 0.031). In contrast, the survival rates were similar between the two treatment groups in the 3- to 5-cm subgroup (RFS, *P* = 0.122; OS, *P* = 0.113). For patients with one recurrent tumor, the combination group had better RFS and similar OS than RFA-alone group (RFS, *P* = 0.001; OS, *P* = 0.094). Meanwhile, for patients with two to three recurrent tumors, the survival rates were similar between the two treatment groups (RFS, *P* = 0.174; OS, *P* = 0.080). In the subgroup of AFP ≤400 μg/L, the combination group was superior to RFA-alone group in terms of both RFA and OS (RFS, *P* = 0.004; OS, *P* = 0.045), whereas the survival rates were similar between the two treatment groups (RFS, *P* = 0.062; OS, *P* = 0.102) in the subgroup of AFP >400 μg/L.

### Re-Recurrence and Treatment

On follow-up, the first re-recurrence occurred in 90 of 103 (87.4%) patients in the combination group, and 103 of 108 (95.4%) patients in RFA-alone group (*P* = 0.038). For the 90 patients with re-recurrence after combined treatment, further treatments aiming at cure were given to 31 patients (34.4%). In the 103 patients with re-recurrence after RFA, such treatments were given to 26 patients (25.2%). The recurrence patterns of re-recurrences were similar between the two groups (**Table 3**). The second and third re-recurrences and the therapies given were summarized in **Supplemental Figure 15**.

**Table 3:** The recurrence pattern of re-recurrences in the two treatment groups.

Recurrence pattern	RFA-Sorafenib	RFA	*P-*value
First recurrence			1.000
Intrahepatic recurrence	87	98	
Extrahepatic recurrence	2	3	
Intrahepatic recurrence + Extrahepatic recurrence	1	2	
Second recurrence			1.000
Intrahepatic recurrence	18	18	
Extrahepatic recurrence	1	0	
Intrahepatic recurrence + Extrahepatic recurrence	2	2	
Third recurrence			0.682
Intrahepatic recurrence	6	3	
Extrahepatic recurrence	1	1	
Intrahepatic recurrence + Extrahepatic recurrence	0	1	

RFA, radiofrequency ablation.

### Adverse Events

No unexpected severe adverse events or treatment-related deaths occurred (**Table 4**). The common adverse events in the two groups were pain, pleural effusion, gastrointestinal bleeding, and fever. There were no significant differences between the two groups. In addition, adverse events that are likely attributable to sorafenib including hand–foot–skin reactions, diarrhea, hypertension, and alopecia were specifically seen in the combination group. These adverse events responded well to conservative treatments.

**Table 4:** Adverse events between the two treatment groups.

Variable	RFA-Sorafenib (n = 103)	RFA (n = 108)	*P*-value
	Grade 1–2/3–4 (%/%)		
Pain	52/2 (50.5/1.9)	59/4 (54.6/3.7)	0.518
Pleural effusion	1/2 (1.0/1.9)	1/1 (0.9/0.9)	1.000
Gastrointestinal bleeding	1/2 (1.0/1.9)	1/1 (0.9/0.9)	1.000
Fever	19/2 (18.4/1.9)	22/0 (20.4/0)	0.233
Hand-foot skin reactions	27/10 (26.2/9.7)	0/0 (0/0)	–
Diarrhea	43/9 (41.7/8.7)	0/0 (0/0)	–
Hypertension	21/4 (20.4/3.9)	0/0 (0/0)	–
Alopecia	18/4 (17.5/3.9)	0/0 (0/0)	–
Nausea/vomiting	48/2 (46.6/1.9)	0/0 (0/0)	–
Fatigue	29/8 (28.2/7.8)	0/0 (0/0)	–
Dysphonia	6/1 (5.8/1.0)	0/0 (0/0)	–
Decreased appetite	45/9 (43.7/8.7)	0/0 (0/0)	–
Pyrexia	18/1 (17.5/1.0)	0/0 (0/0)	–
Rash	22/1 (21.4/1.0)	0/0 (0/0)	–
Weight decreased	19/1 (18.4/1.0)	0/0 (0/0)	–
Headache	8/1 (7.8/1.0)	0/0 (0/0)	–
ALT increased	40/11 (38.8/10.7)	0/0 (0/0)	–
Hyperbilirubinemia	19/2 (18.4/1.9)	0/0 (0/0)	–
Constipation	13/1 (12.6/1.0)	0/0 (0/0)	–
Oral mucositis	15/1 (14.6/1.0)	0/0 (0/0)	–

RFA, radiofrequency ablation; ALT, alanine aminotransferase.

## Discussion

This multicentric study demonstrated that adjuvant sorafenib following RFA provided better survival than RFA alone in patients with early-stage recurrent HCC with MVI at the initial hepatectomy. Moreover, we also found that MVI grade could guide the application of adjuvant sorafenib. In detail, for patients with M1, only patients with a tumor size of 3–5 cm, tumor number of two to three, or AFP >400 μg/L would benefit from the combination therapy, whereas for patients with M2, the combination therapy would be recommended in those with a tumor size of 2–3 cm, one recurrent tumor, or AFP ≤400 μg/L.

The survival advantage of combining RFA with sorafenib for patients with recurrent HCC with MVI at the initial hepatectomy is multifactorial. First, the heat-sink phenomenon compromises RFA-induced tumor necrosis and limits the effectiveness of RFA (21). Recurrent tumors that develop from HCC with MVI positivity are more likely to possess increased angiogenesis due to the aggressive behavior of the initial HCC (6). The anti-angiogenic effect of sorafenib can decrease microvascular density, reduce blood perfusion around the tumor, and thus cause less heat-sink effect, leading to enhanced zones of RFA-induced coagulative necrosis (22, 23). Second, sorafenib can inhibit epithelial–mesenchymal transition of HCC cells following insufficient ablation, thus slowing HCC progression (24). Furthermore, sorafenib can cause enhancement of macrophage number of T cells, thus contributing to delivering an anti-tumor effect on non–RFA-targeted tumor micrometastases (25). These effects of sorafenib probably contributed to the decrease in tumor re-recurrence after RFA and better survival of the combination therapy. Compared with the previous studies on the combination therapy (11–13), this study was a multicentric one and focused on patients with early-stage recurrent HCC with MVI at the initial hepatectomy, pioneering to determine the role of adjuvant sorafenib following RFA in patients with early-stage recurrent HCC with MVI at the initial hepatectomy. The ratios of M1 and M2 were similar to those reported previously (26). Notably, we found that MVI grade could guide the application of adjuvant sorafenib. On the whole, the combination therapy improved both RFS and OS in patients with M2, whereas OS was not improved in patients with M1. To be specific, for patients with M1, only patients with a tumor size of 3–5 cm, tumor number of two to three, or AFP >400 μg/L would benefit from the combination therapy, whereas for patients with M2, the combination therapy would be recommended in those with a tumor size of 2–3 cm, one recurrent tumor, or AFP ≤400 μg/L. M2 grade is associated with higher recurrence rate and worse survival than M1 grade in patients with HCC, possibly due to higher likelihood of residual tumor (26). Therefore, the aforementioned advantages of sorafenib could be fully taken in patients with M2. Likewise, larger tumor size, more tumors, and higher AFP level have also been documented as risk factors for HCC prognosis (27, 28). For patients with HCC with a tumor size of 3–5 cm, tumor number of two to three, or AFP >400 μg/L, it becomes difficult for RFA alone to reach at least 1 cm of safety margin beyond the tumor at every direction (29). Insufficient ablation zone can leave residual tumor in adjacent liver tissues, leading to early recurrence and poor prognosis (30). Therefore, it is necessary to apply adjuvant sorafenib to facilitate RFA in patients with M1 along with a tumor size of 3–5 cm, tumor number of two to three, or AFP >400 μg/L. We also found that the combination therapy was not beneficial in patients with M1 along with a tumor size of 2–3 cm, one recurrent tumor, or AFP ≤400 μg/L, probably because sorafenib was unable to be taken full advantage in this subpopulation. Likewise, the combination therapy may not be recommended for patients with M2 along with a tumor size of 3–5cm, tumor number of two to three, or AFP >400 μg/L because adjuvant sorafenib seemed inadequate to enhance the efficacy of RFA. Therefore, our study provided a hint that not all patients with recurrent HCC with MVI at the initial hepatectomy would benefit from the combination of sorafenib and RFA. Clinicians could apply the combination therapy in a meticulous and precise way with the assistance of MVI grade to avoid unnecessary healthcare burdens and delay in treatment for ineligible patients.

Univariable and multivariable analysis revealed that, in addition to MVI grade and treatment allocation, tumor size, tumor number, PLT, AFP, and interval of recurrence from initial treatment were also independent prognostic factors. Tumor size and number can reflect tumor burden and their prognostic role has been proved in Feng X’s study investigating the role of RFA combined with sorafenib in patients with BCLC stage 0–B1 HCC (11). PLT can facilitate tumor proliferation and metastasis *via* activating the TGFβ/Smad pathway in cancer (31). It has been incorporated into several prognostic indices in predicting HCC survival (32–34). High AFP levels and a short interval of recurrence from initial treatment are associated with aggressiveness and worse survival of HCC (35), and they have been proved to be independent risk factors in patients with early-stage RHCC (3).

There are several limitations to this study. First, as with any retrospective studies, there are the risks of selection and confounding biases. Second, no biopsy was done to confirm recurrence and re-recurrence. However, the noninvasive diagnostic criteria have been shown to achieve high accuracies in many prospective studies (36, 37). Third, the majority of patients had hepatitis B infection in the current study; therefore, the application of this study may be limited in patients with HCC from other etiologies. Fourth, because the combination therapy was included in an aggressive and iterative multimodal management of additional re-recurrences, long-term OS should be evaluated in this context.

In conclusion, adjuvant sorafenib following RFA was associated with better survival than RFA alone in patients with early-stage recurrent HCC with MVI at the initial hepatectomy. Moreover, MVI grade could guide the application of adjuvant sorafenib. More solid evidence from large multicentric prospective studies is necessary to validate these findings.

## Data Availability Statement

The raw data supporting the conclusions of this article will be made available by the authors, without undue reservation.

## Ethics Statement

The studies involving human participants were reviewed and approved by the Ethics Committee of the Anhui Provincial Hospital, the Ethics Committee of the Beijing Cancer Hospital, the Ethics Committee of the Shanghai Eastern Hepatobiliary Surgery Hospital, the Ethics Committee of the Fudan Zhongshan Hospital, the Ethics Committee of the Cancer Center of Sun Yat-sen University, the Ethics Committee of the Bethune First Hospital of Jilin University, the Ethics Committee of the Tianjin Medical University Cancer Hospital, the Ethics Committee of the Xijing Hospital, the Ethics Committee of the Cancer Hospital Chinese Academy of Medical Sciences, the Ethics Committee of the First Affiliated Hospital of Zhejiang University, the Ethics Committee of the First Affiliated Hospital of Zhengzhou University, and the Ethics Committee of the Southwest Hospital of AMU. Written informed consent for participation was not required for this study in accordance with the national legislation and the institutional requirements.

## Author Contributions

M-SC, YC, W-YL, and Z-WP contributed to conception and design of the study. YC and Y-JZ organized the data collection. MC-W and Y-JZ performed the statistical analysis. M-CW and Z-WP wrote the first draft of the manuscript. Y-JZ, M-SC, W-YL, and Z-WP wrote sections of the manuscript. All authors contributed to manuscript revision and approved the submitted version.

## Funding

This study was supported by grants from the National high level talents special support plan— “Ten thousand plan”—Young top-notch talent support program (grant no. not available) and the National Natural Science Foundation of China (Nos. 82072029 and 81770608).

## Conflict of Interest

The authors declare that the research was conducted in the absence of any commercial or financial relationships that could be construed as a potential conflict of interest.

## Publisher’s Note

All claims expressed in this article are solely those of the authors and do not necessarily represent those of their affiliated organizations, or those of the publisher, the editors and the reviewers. Any product that may be evaluated in this article, or claim that may be made by its manufacturer, is not guaranteed or endorsed by the publisher.

## Abbreviations

HCC, hepatocellular carcinoma; RFA, radiofrequency ablation; AFP, alpha-fetoprotein; MVI, microvascular invasion; TACE, transarterial chemoembolization; BCLC, Barcelona Clinic Liver Cancer group; OS, overall survival; ECOG, Eastern Cooperative Oncology Group; CEUS, contrast-enhanced ultrasound; CECT, contrast-enhanced computed tomography; RFS, recurrence-free survival; HBsAg, hepatitis B surface antigen; PLT, platelet; ALB, albumin; ALT, alanine aminotransferase; TBIL, total bilirubin; HR, hazard ratio; CI, confidence interval.
