# Supplementary material for: Adjuvant Sorafenib Following Radiofrequency Ablation for Early-Stage Recurrent Hepatocellular Carcinoma With Microvascular Invasion at the Initial Hepatectomy
Source: Front Oncol. 2022 Jun 23;12:868429. doi: 10.3389/fonc.2022.868429 (PMC9260661; doi:10.3389/fonc.2022.868429)
Supplement: Supplementary file 1 [file DataSheet_1.docx]

Supplementary Material

## Supplementary Figures


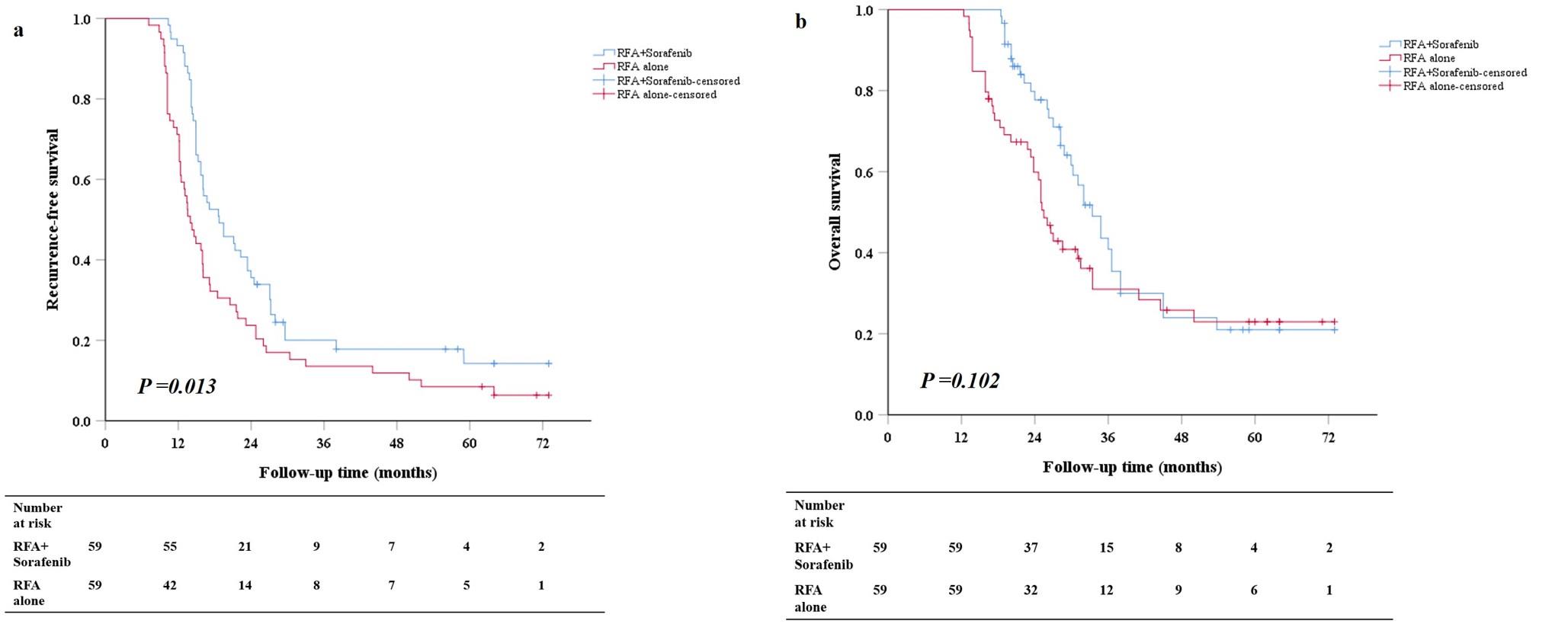


**Supplemental Figure 1** Cumulative survival curves of RFS (a) and OS (b) between the combination group and RFA alone group in patients with M1. Abbreviations: RFS, recurrence-free survival; OS, overall survival; RFA, radiofrequency ablation.


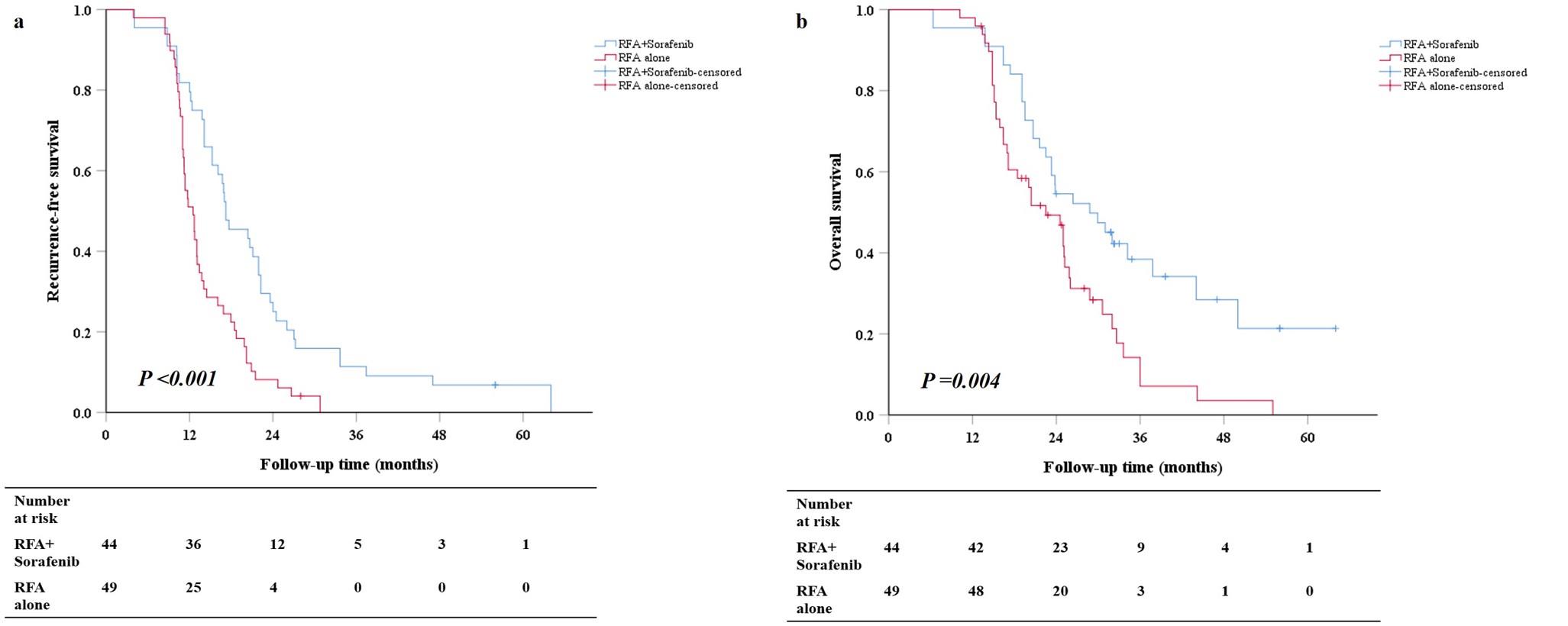


**Supplemental Figure 2** Cumulative survival curves of RFS (a) and OS (b) between the combination group and RFA alone group in patients with M2. Abbreviations: RFS, recurrence-free survival; OS, overall survival; RFA, radiofrequency ablation.


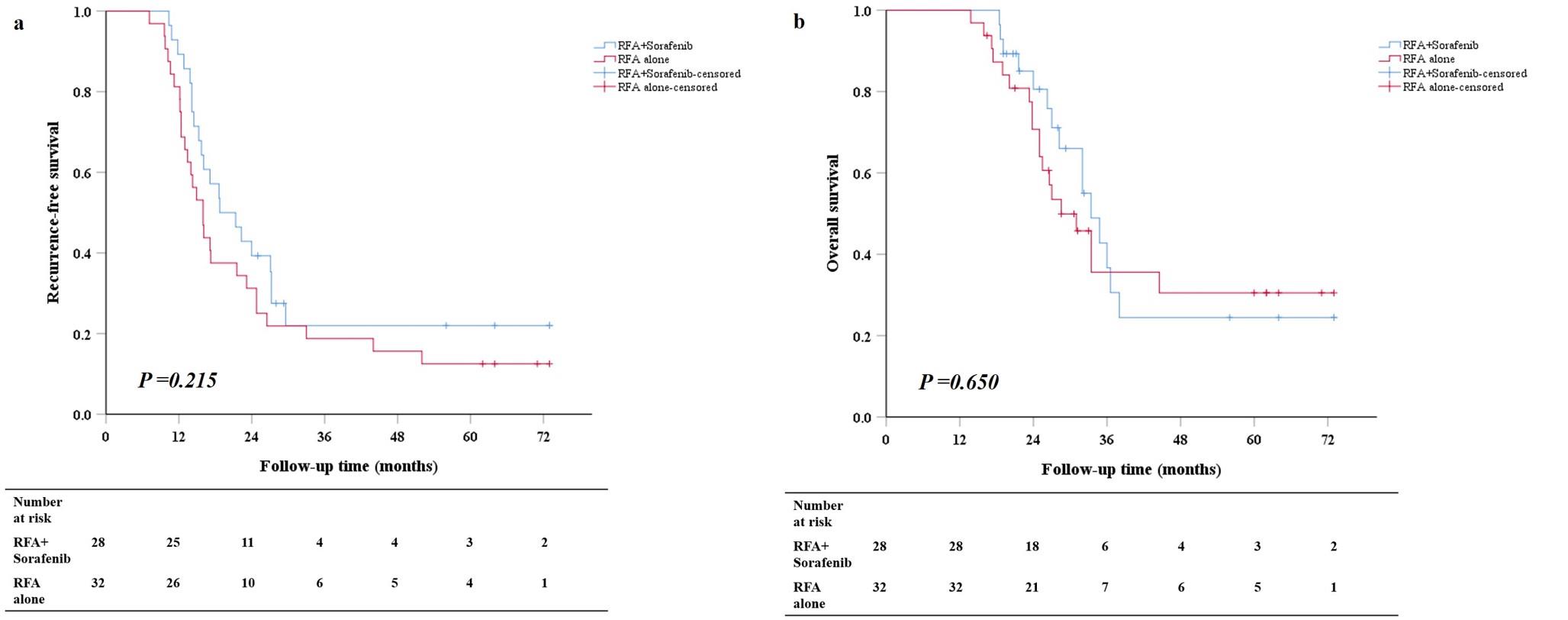


**Supplemental Figure 3** Cumulative survival curves of RFS (a) and OS (b) between the combination group and RFA alone group in patients with M1 and a tumor size of 2-3cm. Abbreviations: RFS, recurrence-free survival; OS, overall survival; RFA, radiofrequency ablation.


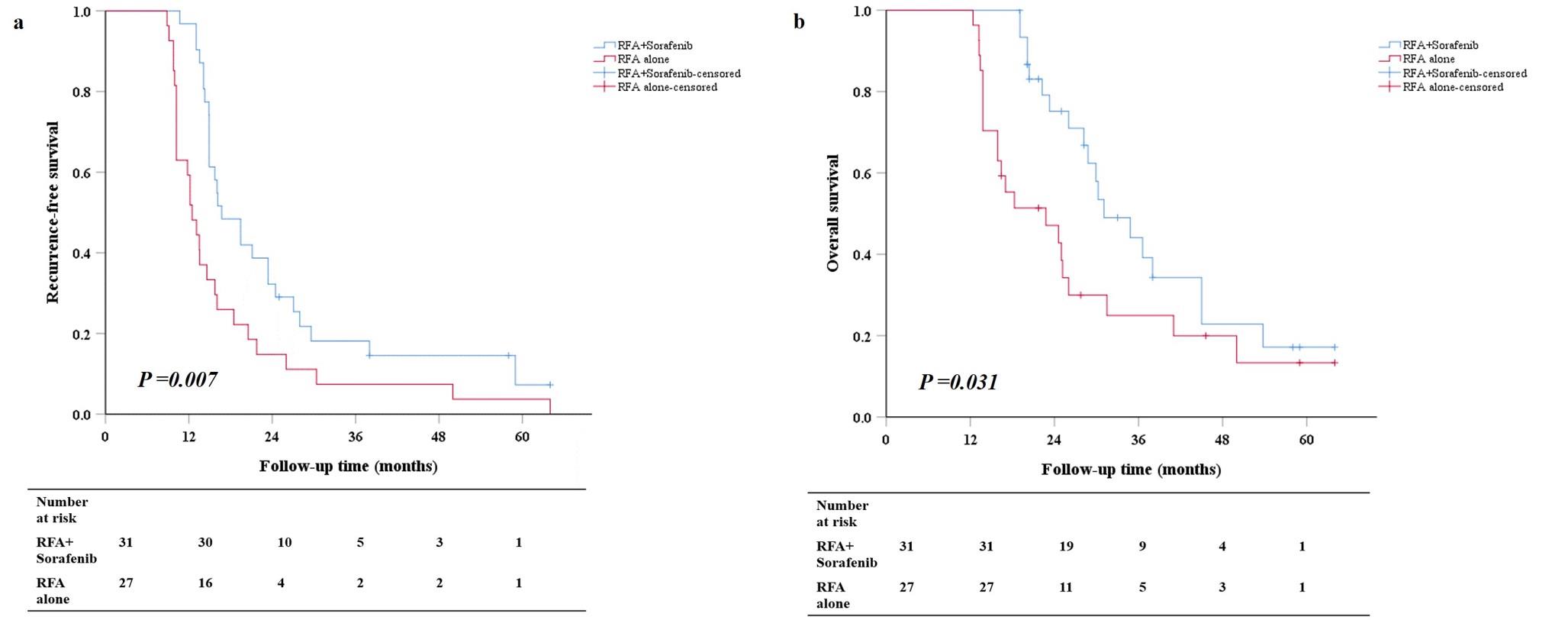


**Supplemental Figure 4** Cumulative survival curves of RFS (a) and OS (b) between the combination group and RFA alone group in patients with M1 and a tumor size of 3-5cm. Abbreviations: RFS, recurrence-free survival; OS, overall survival; RFA, radiofrequency ablation.


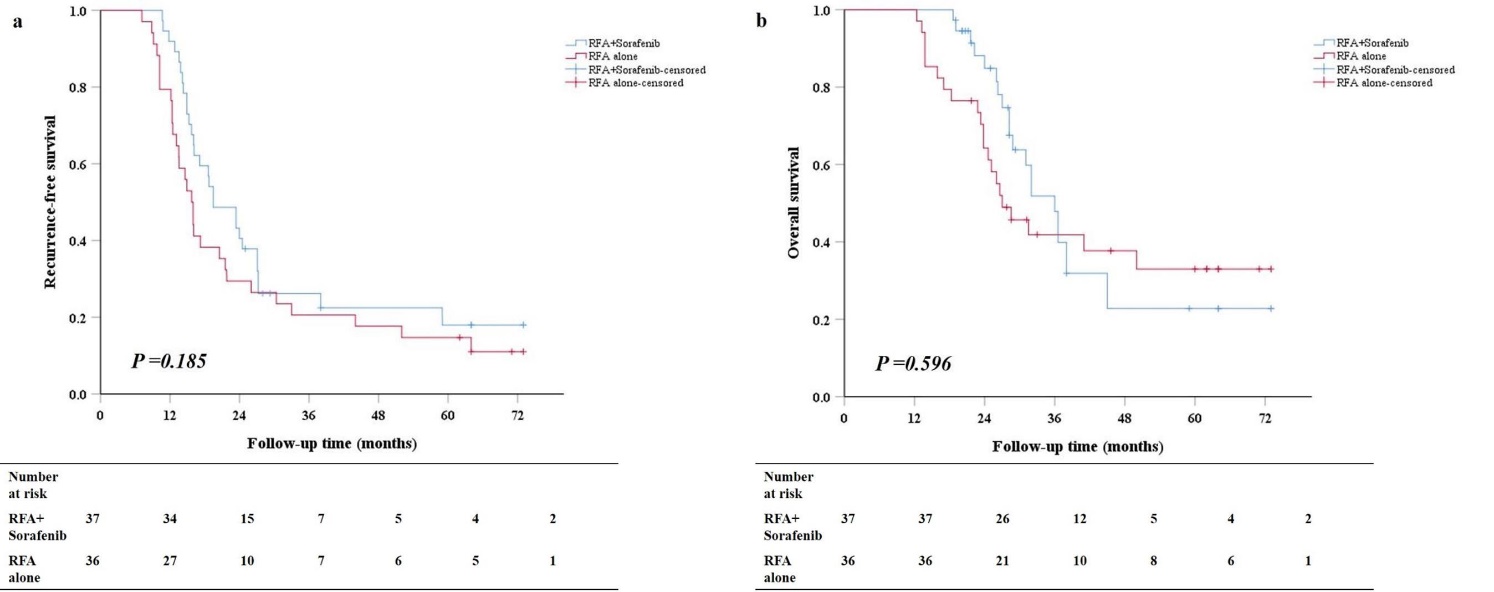


**Supplemental Figure 5** Cumulative survival curves of RFS (a) and OS (b) between the combination group and RFA alone group in patients with M1 and 1 recurrent tumor lesion. Abbreviations: RFS, recurrence-free survival; OS, overall survival; RFA, radiofrequency ablation.


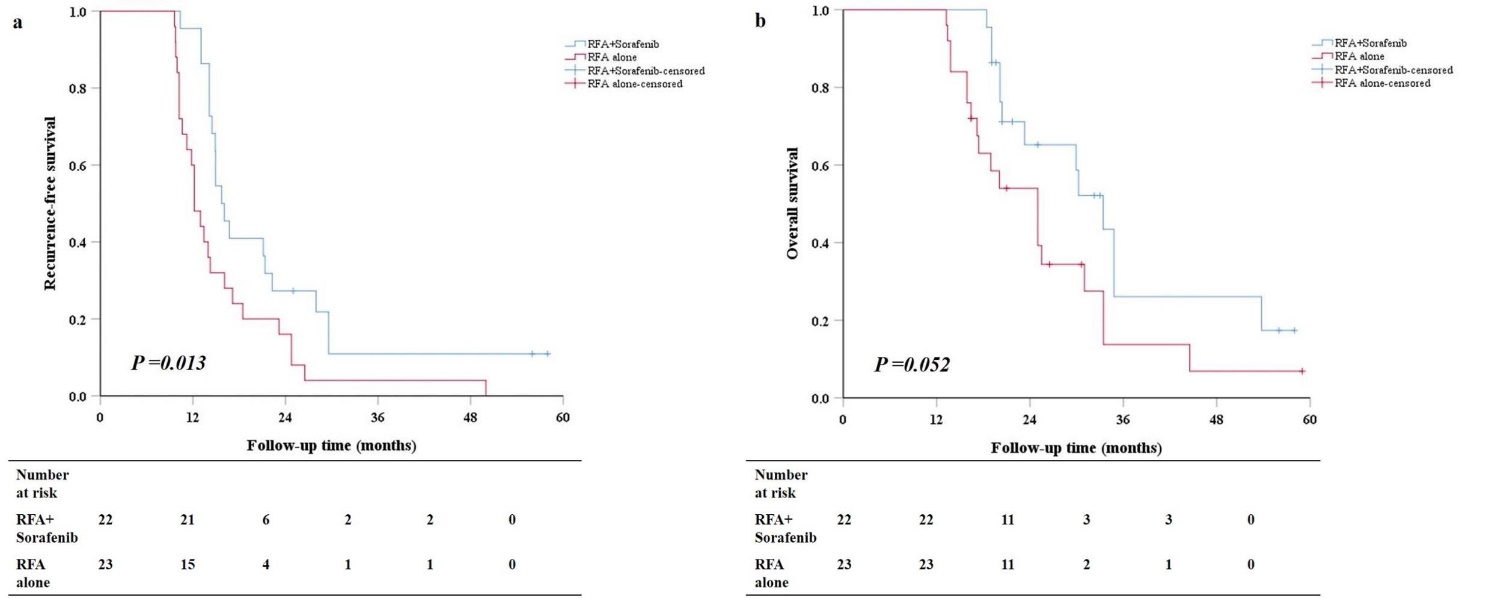


**Supplemental Figure 6** Cumulative survival curves of RFS (a) and OS (b) between the combination group and RFA alone group in patients with M1 and 2-3 recurrent tumor lesions. Abbreviations: RFS, recurrence-free survival; OS, overall survival; RFA, radiofrequency ablation.


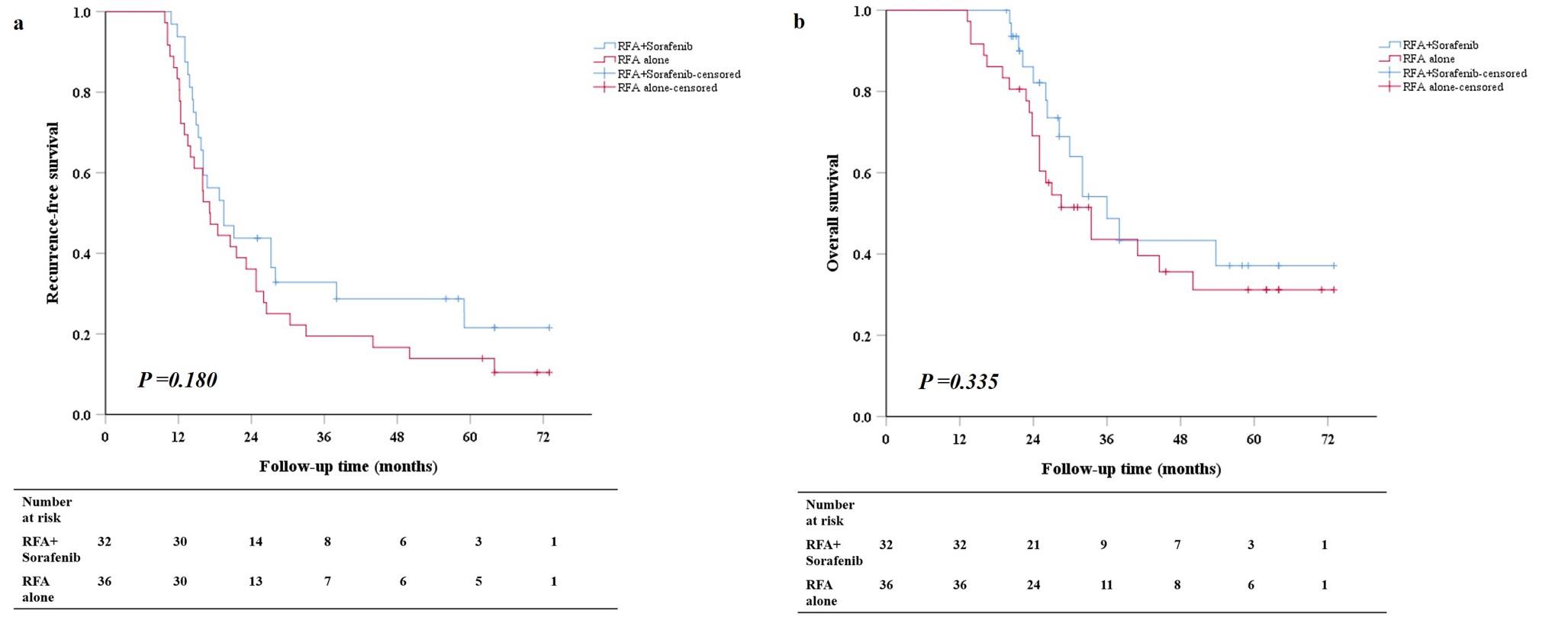


**Supplemental Figure 7** Cumulative survival curves of RFS (a) and OS (b) between the combination group and RFA alone group in patients with M1 and AFP ≤ 400μg/L. Abbreviations: RFS, recurrence-free survival; OS, overall survival; RFA, radiofrequency ablation; AFP, alpha-fetoprotein.


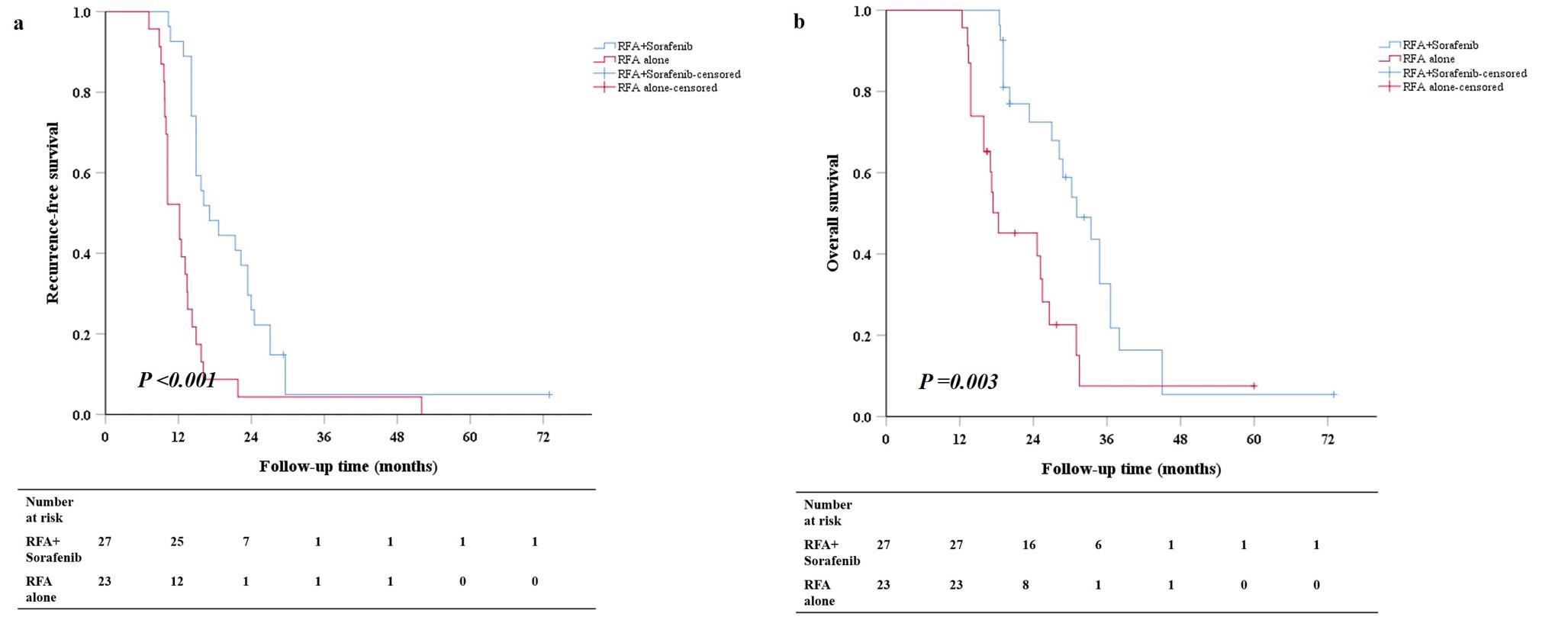


**Supplemental Figure 8** Cumulative survival curves of RFS (a) and OS (b) between the combination group and RFA alone group in patients with M1 and AFP > 400μg/L. Abbreviations: RFS, recurrence-free survival; OS, overall survival; RFA, radiofrequency ablation; AFP, alpha-fetoprotein.


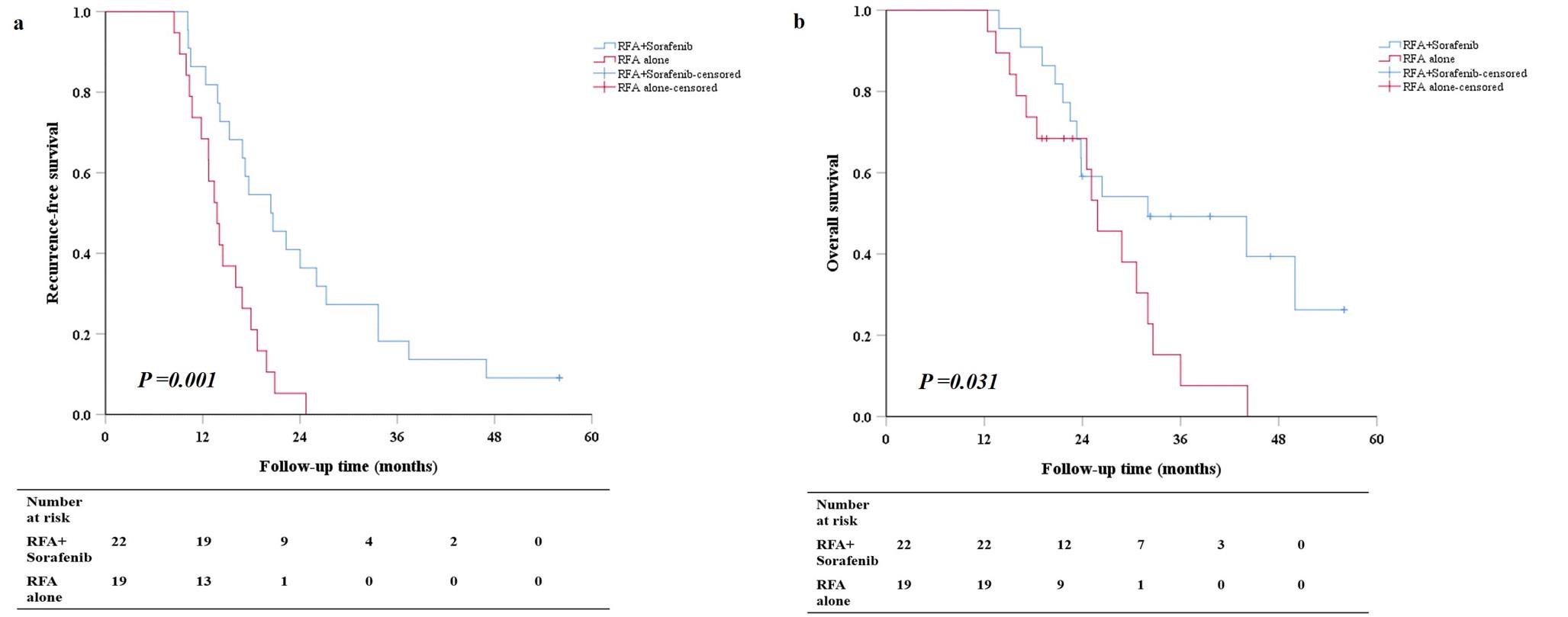


**Supplemental Figure 9** Cumulative survival curves of RFS (a) and OS (b) between the combination group and RFA alone group in patients with M2 and a tumor size of 2-3cm. Abbreviations: RFS, recurrence-free survival; OS, overall survival; RFA, radiofrequency ablation.


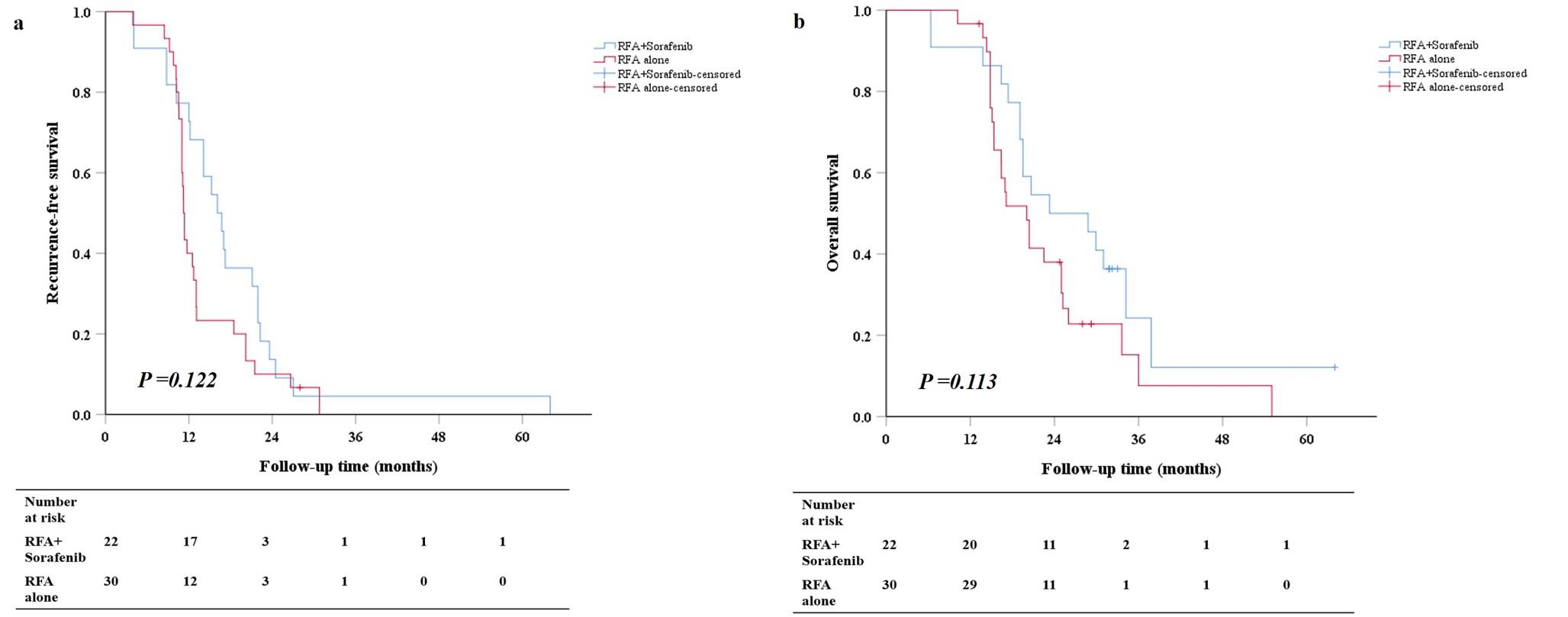


**Supplemental Figure 10** Cumulative survival curves of RFS (a) and OS (b) between the combination group and RFA alone group in patients with M2 and a tumor size of 3-5cm. Abbreviations: RFS, recurrence-free survival; OS, overall survival; RFA, radiofrequency ablation.


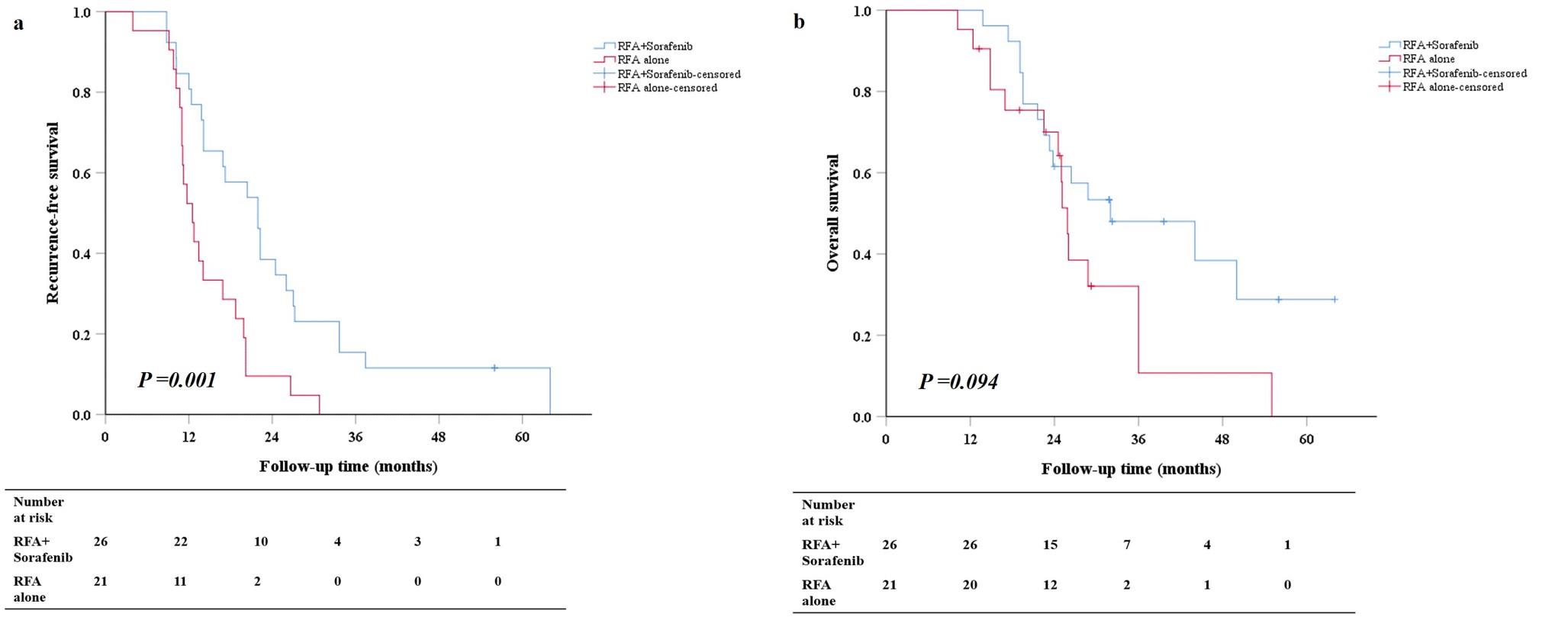


**Supplemental Figure 11** Cumulative survival curves of RFS (a) and OS (b) between the combination group and RFA alone group in patients with M2 and 1 recurrent tumor lesion. Abbreviations: RFS, recurrence-free survival; OS, overall survival; RFA, radiofrequency ablation.


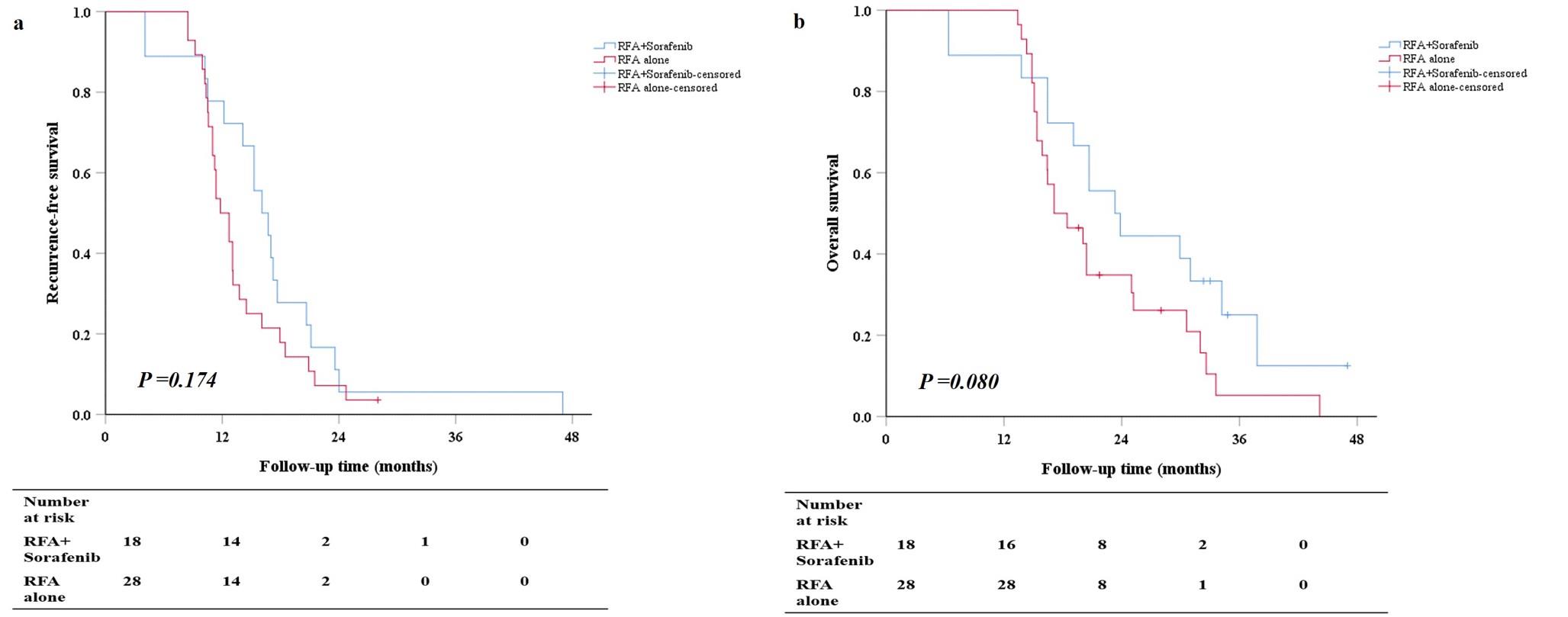


**Supplemental Figure 12** Cumulative survival curves of RFS (a) and OS (b) between the combination group and RFA alone group in patients with M2 and 2-3 recurrent tumor lesions. Abbreviations: RFS, recurrence-free survival; OS, overall survival; RFA, radiofrequency ablation.


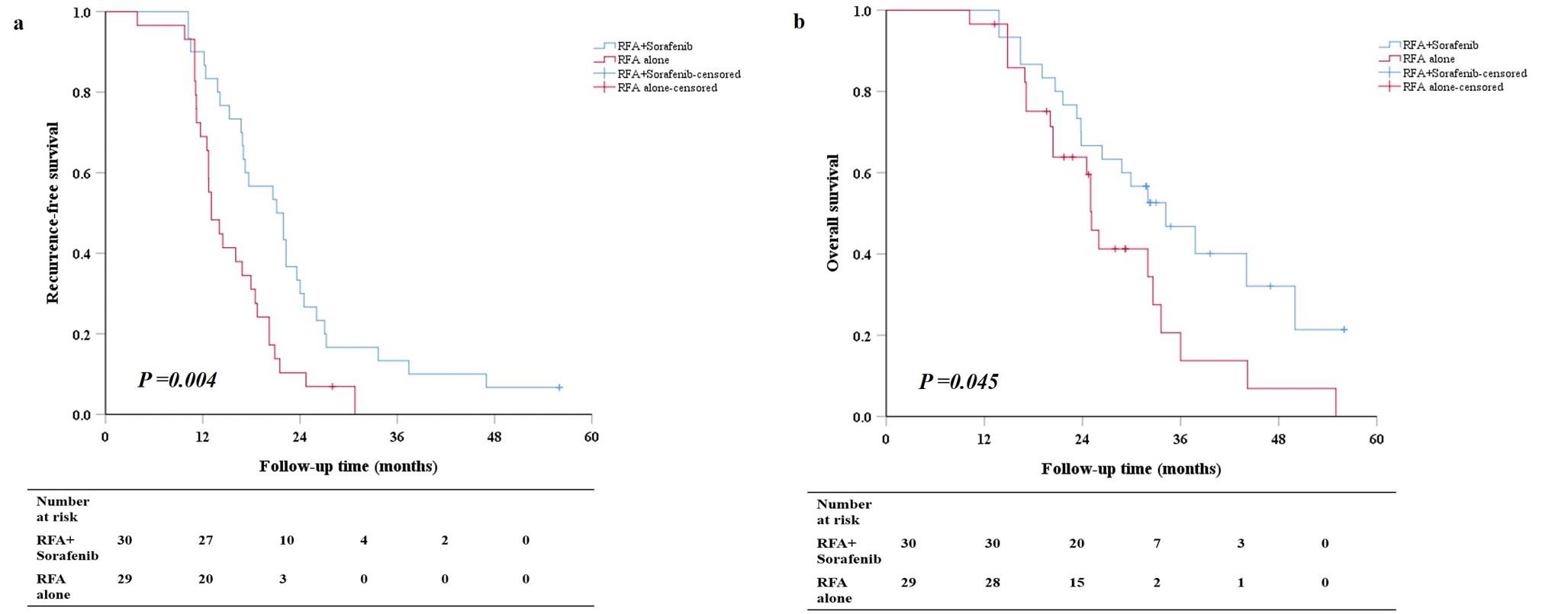


**Supplemental Figure 13** Cumulative survival curves of RFS (a) and OS (b) between the combination group and RFA alone group in patients with M2 and AFP ≤ 400μg/L. Abbreviations: RFS, recurrence-free survival; OS, overall survival; RFA, radiofrequency ablation; AFP, alpha-fetoprotein.


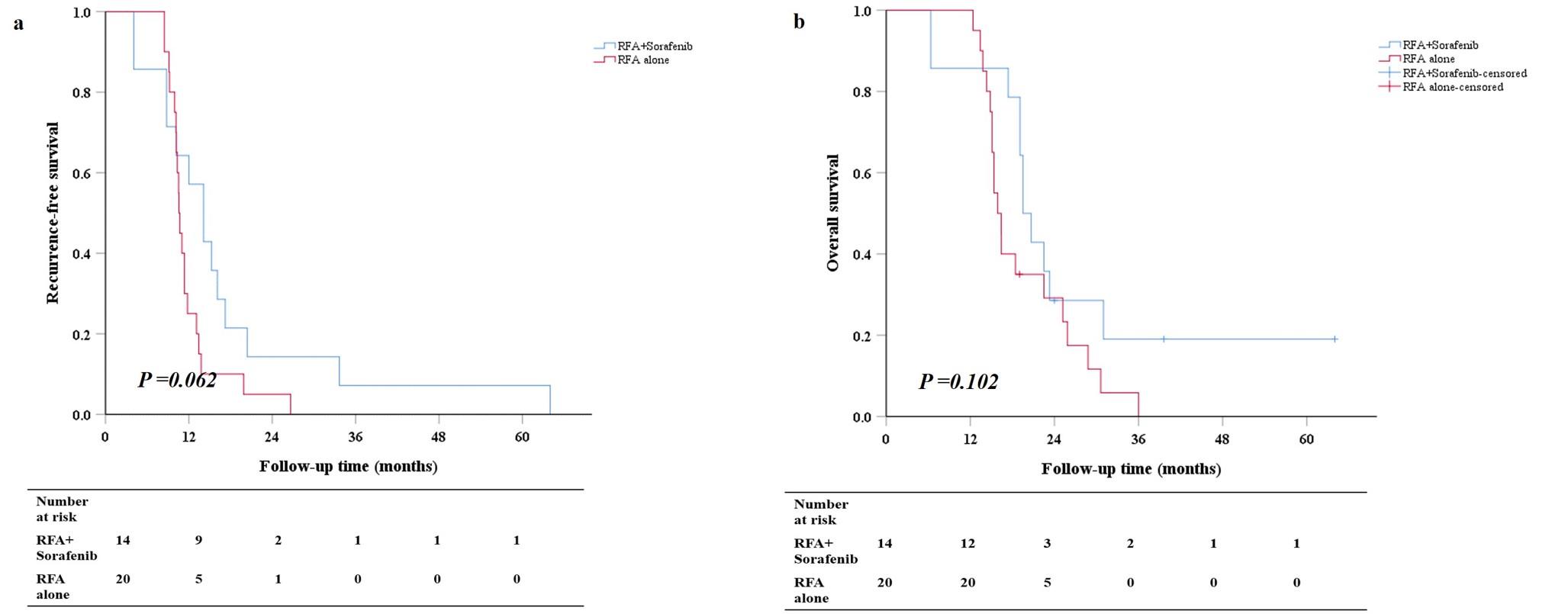


**Supplemental Figure 14** Cumulative survival curves of RFS (a) and OS (b) between the combination group and RFA alone group in patients with M2 and AFP > 400μg/L. Abbreviations: RFS, recurrence-free survival; OS, overall survival; RFA, radiofrequency ablation; AFP, alpha-fetoprotein.


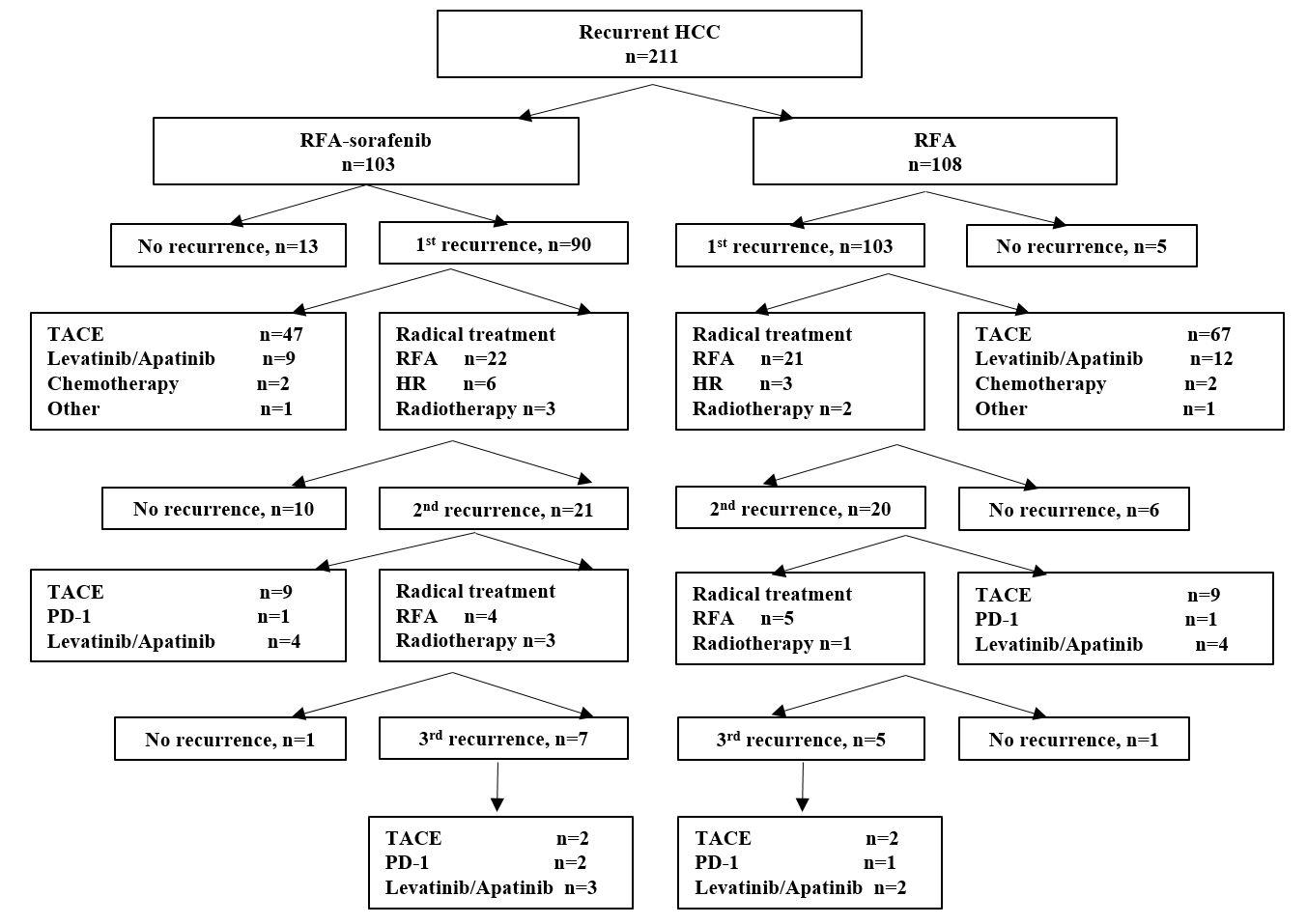


**Supplemental Figure 15** Subsequent further re-recurrences and treatment modalities given in the two treatment groups. Abbreviations: HCC, hepatocellular carcinoma; RFA, radiofrequency ablation; TACE, transarterial chemoembolization; HR, hepatic resection; PD-1, programmed death-1.
